# Scaling up HIV self-testing in sub-Saharan Africa: a review of technology, policy and evidence

**DOI:** 10.1097/QCO.0000000000000426

**Published:** 2018-01-04

**Authors:** Pitchaya P. Indravudh, Augustine T. Choko, Elizabeth L. Corbett

**Affiliations:** a Malawi-Liverpool-Wellcome Trust Clinical Research Programme, Blantyre, Malawi; b Department of Infectious Disease Epidemiology, London School of Hygiene and Tropical Medicine; c Department of Clinical Research, London School of Hygiene and Tropical Medicine, London, England, UK

**Keywords:** HIV self-testing, HIV testing, sub-Saharan Africa

## Abstract

**Purpose of review:**

HIV self-testing (HIVST) can provide complementary coverage to existing HIV testing services and improve knowledge of status among HIV-infected individuals. This review summarizes the current technology, policy and evidence landscape in sub-Saharan Africa and priorities within a rapidly evolving field.

**Recent findings:**

HIVST is moving towards scaled implementation, with the release of WHO guidelines, WHO prequalification of the first HIVST product, price reductions of HIVST products and a growing product pipeline. Multicountry evidence from southern and eastern Africa confirms high feasibility, acceptability and accuracy across many delivery models and populations, with minimal harms. Evidence on the effectiveness of HIVST on increased testing coverage is strong, while evidence on demand generation for follow-on HIV prevention and treatment services and cost-effective delivery is emerging. Despite these developments, HIVST delivery remains limited outside of pilot implementation.

**Summary:**

Important technology gaps include increasing availability of more sensitive HIVST products in low and middle-income countries. Regulatory and postmarket surveillance systems for HIVST also require further development. Randomized trials evaluating the effectiveness and cost-effectiveness under multiple distribution models, including unrestricted delivery and with a focus on linkage to HIV prevention and treatment, remain priorities. Diversification of studies from west and central Africa and around blood-based products should be addressed.

## INTRODUCTION

Adult HIV incidence has largely remained static in sub-Saharan Africa, with an estimated 25.5 million people living with HIV (PLHIV) and 1.3 million new infections annually [1]. Despite increased availability of provider-initiated and community-based HIV testing services (HTS), only 76% of PLHIV in eastern and southern Africa and 42% of PLHIV in western and central Africa are aware of their serostatus [2].

HIV self-testing (HIVST), a process in which individuals collect their own specimen, perform the test and interpret the results, can provide complementary coverage to standard HTS and reach undiagnosed PLHIV and individuals with high ongoing HIV risk [3]. Reactive results need to be confirmed through additional testing by a trained provider, whereas nonreactive results should prompt linkage to prevention services, including voluntary medical male circumcision (VMMC) and preexposure prophylaxis (PrEP) if indicated [3].

The field of HIVST is rapidly evolving. This review summarizes the current technology, policy and evidence landscape for HIVST in sub-Saharan Africa, with priorities for scaled implementation outlined. Current developments in technology and policy were assessed through HIVST.org, a relational map hosted by WHO [4]. Recent and ongoing observational studies and trials were identified through PubMed, ClinicalTrials.gov, ISRCTN and the Pan African Clinical Trials Registry. The review was conducted through September 2017. 

**Box 1:**
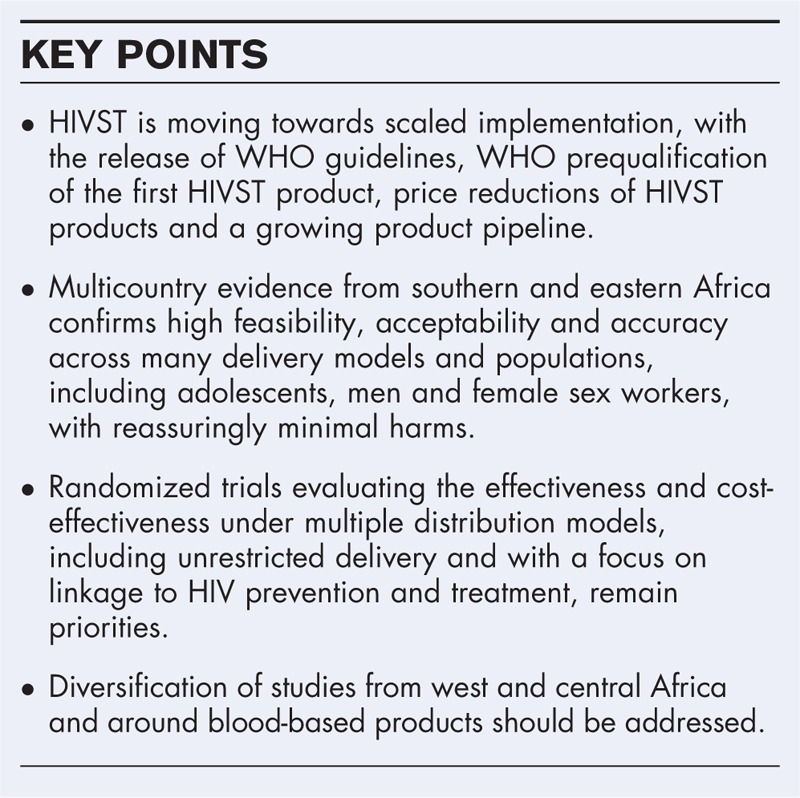
no caption available

## TECHNOLOGY UPDATE

Products for HIV self-testing available in sub-Saharan Africa are listed in Table 1. Products use either oral-fluid or finger-prick blood samples and take between 5 and 7 steps and 1 and 45 minutes to provide results [5]. Ideal products should be easy to use and interpret for optimized accuracy, with clear instructions-for-use (IFU) that are available in local languages and understandable at low literacy and education levels [6]. Most HIVST products in development are repurposed professional-use rapid diagnostic tests (RDTs) [5], with innovation limited to packaging and IFU modifications and single-use parts (e.g. pipette, lancet, etc.) [7]. Most commercially available RDTs for self-testing are second-generation assays, which detect immunoglobin G antibodies but not immunoglobin M antibodies (third generation) nor viral antigens (fourth generation) and nucleic acids. Compared to later generations, second-generation tests require a longer window period of 28 days between infection and test positivity [5].

**Table 1:** HIV rapid diagnostic tests for self-testing available in sub-Saharan Africa

				Pricing in LMIC (US$)	
Name (generation), manufacturer	Specimen	Regulatory approvals	Private sector availability in SSA	Ex-works	Retail
Amethyst HIV 1&2 Test Kit, MYSP Nigeria Ltd.	Oral fluid	NAFDAC	Nigeria		$16
Atomo HIV Self-Test (3rd), Atomo Diagostics	Blood	CE marked	Kenya and South Africa	$3^*^, based on volume	$13.40
autotest VIH (2nd), AAZ-LMB	Blood	CE marked			
BioSURE HIV Self-Test (2nd), BioSure Ltd.	Blood	CE marked		$5	
INSTI HIV Self-Test (3rd), bioLytical Laboratories Inc.	Blood	CE mark pending for modified LMIC product	Kenya	$3	$8–10
OraQuick In-Home HIV Test (2nd), OraSure Technologies Inc.	Oral fluid	FDA			
OraQuick HIV Self-Test (2nd), OraSure Technologies Inc.	Oral fluid	WHO PQ	Kenya and South Africa	$2 for 50 LMIC	$9.50

Adapted from the WHO/Unitaid Market and Technology Landscape: HIV Rapid Diagnostic Tests for Self-Testing. CE, European Conformity; FDA, U.S. Food and Drug Administration; LMICs, low-income and middle-income countries; NAFDAC, Nigeria National Agency for Food and Drug Administration Control; PQ, prequalified; SSA, Sub-Saharan Africa.

In low and middle-income countries (LMIC), price per self-test currently ranges from US$2 to 3 for public sector procurement and US$8–16 retail in the private sector [5]. In 2017, unit costs for OraQuick HIV Self-Test were reduced under a temporary donor agreement, enabling government or charitable purchase for US$2 in 50 LMICs [8]. This agreement has important implications for HIVST market development, with potential for price reductions to increase demand and facilitate a competitive market for HIVST. Alternatively, it could discourage manufacturers from investment in innovations that improve usability and precision but increase costs, underpinning the need for incentives to further product development [7].

## POLICY UPDATE

**FIGURE 1:**
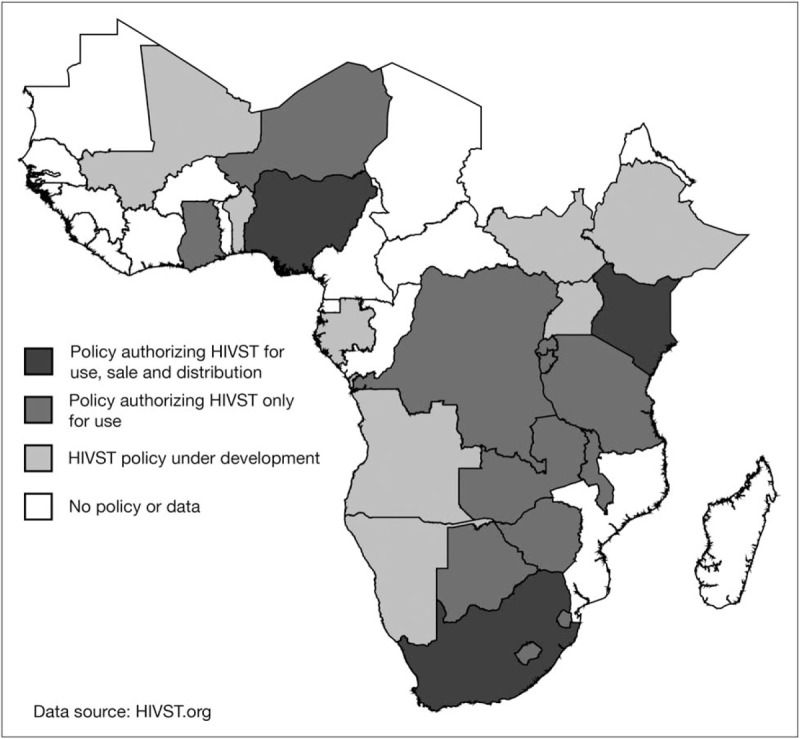
Policy map of HIV self-testing (HIVST) in sub-Saharan Africa.

International and national policy to guide HIVST implementation, as well as regulatory and quality assurance systems, are integral to scale-up in sub-Saharan Africa. In 2016, WHO released guidelines recommending HIVST based on evidence of increased uptake and frequency of testing, especially among underserved and high-risk populations [3]. This prompted a shift in national policies supportive of HIVST, from four countries in 2015 to 40 countries at the time of the review, of which 15 countries are from sub-Saharan Africa (Fig. 1) [9]. Despite the changing policy environment, few countries are implementing HIVST at scale and only Kenya has released full operational guidelines [10].

Regional and national regulatory systems for HIVST are also generally poorly developed in sub-Saharan Africa, with unregulated and low-quality products available for purchase in certain markets [11]. To inform procurement decisions, sub-Saharan African countries tend to rely on approvals issued from founding members of the Global Harmonization Task Force or WHO [5], which prequalified its first HIVST product for LMICs in 2017 [12]. Approved products are also now available through limited pharmacies and retailers in Kenya and South Africa [13,14]. However, given the numerous potential outlets for HIVST through the private sector, countries will need to reassess their regulatory and postmarket surveillance systems to ensure adequate consumer protection from ineffective devices, without presenting undue barriers to bona fide manufacturers.

## REVIEW OF EVIDENCE

### Readiness and preferences for HIV self-testing

Successful implementation of HIVST hinges on both provider readiness to deliver HIVST and public demand once HIVST services have been established. Early studies in sub-Saharan Africa reported high interest in HIVST among the general population [15–17], couples [18], high-risk populations [19], healthcare providers [20–22] and policy stakeholders [23,24]. Recent evidence confirms high readiness to self-test among a wider range of populations [25,26^▪^,27^▪^,^28^–^34^,^35^^▪▪^,^36^^▪▪^,^37^,^38^], notably men [26^▪^,^32^], young people [27^▪^,^33^,^36^^▪▪^], serodiscordant couples [35^▪▪^], and sex workers and their partners [25,30] (Table 2). Principal motivations for self-testing include convenience and associated time and cost savings; control over the testing process; privacy and confidentiality; and ease-of-use and painlessness of the oral fluid-based self-test [17,18,21–25,26^▪^,27^▪^,^28^,^30^–^32^,^34^,^35^^▪▪^,^37^,^38^]. Nearly, all of these studies evaluated oral fluid tests, although two South African studies showed high acceptability of blood-based products [36^▪▪^,^38^].

**Table 2:** Recent observational studies and randomized trials on HIV self-testing, 2016–2017

Study	Location	Design	Population	Values	Preferences	Uptake and linkage	Costs	Social/behavioral impact	Performance and usability
Burke *et al.*2017	Uganda	FGDs and IDIs (*n* = 88)	General population, high-risk fishing populations and HCWs in rural areas	High support for HIVST, but concerns around absence of HCWs	Preference for obtaining HIVST from health facilities. Young men preferred lodges and bars. Willingness to pay ranged from US$0.29 to $29				
Choko *et al.* 2016	Malawi	Quality assurance study of OFST (*n* = 378)	Urban general population						Kappa of 0.97 between preincubated and optimally stored OraQuick. Visual stability retained over 1 year for 1 of 375 preincubated and 1 of 371 optimally stored tests
Choko *et al.* 2017	Malawi	FGDs and IDIs (*n* = 62)	Women attending ANC in urban areas and male partners	Strong interest in providing HIVST kits for delivery to male partners, with low potential for IPV	Preferences for fixed financial incentives (US$3 or US$10) and phone call reminders to support linkage				
Indravudh *et al.* 2017	Malawi, Zimbabwe	DCEs (*n* = 341)FGDs and IDIs, and provision of assisted OFST (*n* = 122)	Young people (16–25 years) in rural areas	High willingness to self-test, valuing enhanced discretion and autonomy	Strongest preferences for home delivery and free kits, followed by community distributors and some in-person support				
Kelvin *et al.* 2016	South Africa	FGDs (*n* = 20)	Urban general population	HIVST seen to remove barriers to standard HTS and facilitate partner testing. Concerns included lack of HCWs					
Kelvin *et al.* 2017	Kenya	Cross-sectional survey, and offer of assisted OFST, standard HTS or no testing at health facilities (*n* = 149)	Male truck drivers			56.4% accepted HIVST at health facilities. 23.5% accepted standard HTS			13.1% required unsolicited correction. Errors included difficulties in reading results
Knight *et al.* 2017	South Africa	Cross-sectional survey and interviews, and provision of either unassisted OFST or BST (*n* = 50)	Rural and peri-urban general population	High interest in HIVST. Emphasized need for clear information on testing and linkage process	Preference for delivery at health facilities and private sector outlets. Willingness to pay ranged from ZAR10 to 150				High perceived ease of use. Difficulties around use of lancet with BST
Kurth *et al.* 2016	Kenya	Cross-sectional survey, and provision of unassisted OFST (*n* = 240)	General population	94% reported HIVST was acceptable	Mean willingness to pay of US$1.25. Lower for women and young people				Kappa of 0.89 between OraQuick and ELISA. 89.7% sensitivity, 98.0% specificity, 15% invalid results. High reported ease of use (95.4%). Errors included incorrect use of swab
Maheswaran *et al.* 2016	Malawi	Costing study of community-based delivery of OFST and facility HTS (*n* = 1291)	Urban general population				Provider ($8.78) and user ($0.00) unit costs per test were lower, but provider costs per positive test ($97.50) were higher, for HIVST compared to facility HTS		
Maheswaran *et al.* 2017	Malawi	Costing study of the first year of ART after OFST and facility HTS (*n* = 325)	Urban general population				No differences between HIVST and facility HTS for provider and societal costs per person initiated on ART and the first year of ART		
Maman *et al.* 2017	Kenya	IDIs, and provision of assisted OFST for secondary distribution (*n* = 18)	FSWs in urban areas	Enthusiasm around self-testing		Most distributed kits to primary partners or regular commercial sex clients		Partners receiving self-tests intentionally selected to minimize social harms.HIVST sometimes used for point-of-sex decision-making	
Martinez Perez *et al.* 2016	South Africa	FGDs and IDIs (*n* = 25)	Urban general population	Delivery of HIVST from health facilities for home use was highly acceptable. Concerns included absence of HCWs					
Martinez Perez *et al.* 2016	South Africa	Cross-sectional survey, and provision of assisted OFST (*n* = 2198)	Rural general population						Kappa of 0.993 between OraQuick and provider-delivered HTS. 98.7% sensitivity, 100% specificity. User error rate of 0.09%. Errors included spillage of developer fluid
Masters *et al.* 2016	Kenya	RCT with allocation to secondary distribution of assisted OFST, or invitation letter for clinic-based testing, for male partners. (*n* = 570)	Women attending ANC or PPC in urban areas			Partner testing (90.8 versus 51.7%), couples testing (75.4 versus 33.2%) and knowledge of partner status (89.8 versus 59.7%) were more likely for HIVST than SOC. Linkage for partners with reactive results was 2 of 8 for HIVST and 3 of 4 for SOC		No incidence of IPV reported	95% reported partners found HIVST to be easy
Matovu *et al.* 2017	Uganda	FGDs and IDIs (*n* = 92)	Women attending ANC and male partners	Secondary delivery of HIVST kits to partners viewed positively. Minimal concerns regarding IPV in steady relationships					
Mokgatle *et al.* 2017	South Africa	Cross-sectional survey (*n* = 3662)	Tertiary students	87.1% indicated HIVST was acceptable	Preference for pretest counseling using instruction leaflets (47.9%) and posttest counseling using hotlines (40.0%). 74.7% willing to buy self-tests				
Mugo *et al.* 2017	Kenya	Cross-sectional survey, and offer of assisted OFST sold at US$1 at pharmacies (*n* = 463)	Pharmacy clients and service providers in urban areas	94% agreed HIVST kits should be available in pharmacies	96% preferred to access HIVST at pharmacies	35% bought self-tests, with uptake higher among clients seeking services related to HIV-risk (84%). 66% took the kits home			
Ngure *et al.* 2017	Kenya	Cross-sectional survey, FGDs and IDIs, and offer of assisted OFST at PrEP clinics (*n* = 226)	HIV-uninfected adults in sero-discordant couples on PrEP	High interest in HIVST for use in between clinic testing while on PrEP	56.7% preferred OFST to provider-delivered HTS	98% accepted HIVST kits, with 95.6% of 1282 kits used. 67.7% self-tested alone		No social harms reported	96.8% reported HIVST was easy. 90.8% did not require help to test
Smith *et al.* 2016	South Africa	Cross-sectional survey and provision of assisted BST (*n* = 224)	Young people (16–24 years) in urban areas	Mean acceptability score was 4.3/5. Higher for younger people and debut testers	74.9% preferred the BST to provider-delivered HTS				96.4% correctly completed the test and interpreted results. Mean usability score was 4/5. Errors included insufficient specimen collection
Spyrelis *et al.* 2017	South Africa	FGDs (*n* = 118)	Urban general population	High willingness to self-test. Absence of HCW was a disadvantage for men					
Thirumurthy *et al.* 2016	Kenya	Longitudinal cohort study, and secondary distribution of assisted OFST (*n* = 280)	Women attending ANC or PPC and FSWs in urban areas			75–91% distributed to primary sex partners, with high rates of couples testing. 80% of FSWs also distributed to clients. 99% of kits given to sexual partners were used. Linkage for partners with reactive results was 2 of 4 for ANC or PPC clients and 26 of 51 for FSWs		Higher proportions of women had sexual intercourse (62 versus 18%) and used condoms (44 versus 100%) when partners had nonreactive versus reactive results. Four participants reported IPV	

Assisted HIV self-testing refers to individuals who receive in-person guidance or demonstration on how to self-test before or during the procedure. ANC, antenatal care; ART, antiretroviral therapy; BST, blood-based self-test; DCEs, discrete choice experiments; FGDs, focus group discussions; FSWs, female sex workers; HCWs, healthcare workers; HIVST, HIV self-testing; HTSs, HIV testing services; IDIs, in-depth interviews; IPV, intimate partner violence; OFST, oral fluid-based self-testing; PPC, postpartum care; PrEP, preexposure prophylaxis; RCT, randomized controlled trial; SOC, standard of care; ZAR, South African Rand.

Specific values and preferences for HIVST varied by population group. In Kenya, there was high interest among the general population for fee-based, pharmacy models because of enhanced accessibility [34]. Alternatively, individuals who valued access to professionalized care preferred to obtain HIVST kits from health facilities [25,31]. Young people in Malawi and Zambia preferred low-cost, home delivery and valued HIVST for providing greater discretion around their sexual debut [27^▪^]. Men expressed interest in distribution through lodges and bars because of flexible service hours amid their work obligations [25]. Secondary distribution of self-tests to male partners of antenatal care clients similarly provided added convenience and privacy compared to clinic-based couples testing, wherein male attendance was often stigmatized [26^▪^]. Sex workers valued HIVST as a way to demonstrate trust to primary partners and inform sexual decision-making with commercial sex clients [30], whereas HIV-uninfected individuals on PrEP found that HIVST reduced anxiety in between scheduled testing at clinics [35^▪▪^]. Willingness to purchase HIVST kits varied across studies (Table 2) [19,22,24,25,27^▪^,^28^,^29^,^33^,^34^,^38^], with low ability to pay reported among women and young people [27^▪^,^29^].

Commonly reported concerns included the possibility for coercive testing and inability of individuals to self-test accurately, psychologically cope with reactive results and successfully link to confirmatory testing and care [17,20,23–25,26^▪^,27^▪^,^28^,^31^,^34^,^37^,^38^]. User-suggested solutions to tackle potential adverse effects included clear, locally translated IFUs, coupled with development of effective training and educational materials for providers and accessible in-person and hotline-based support for self-testers [25,27^▪^,^31^,^33^,^37^,^38^]. A recent study among Malawian men found that time-limited behavioral motivators, specifically financial incentives (US$3 and $10) and phone call reminders, were also acceptable to facilitate linkage to prevention and care [26^▪^].

### Performance and usability of HIV self-testing

The public health impact of HIVST is contingent on user ability to self-test and confidence in the results to access further HIV services. Blood-based self-tests have generally performed higher than oral fluid-based self-tests, with sensitivity of 96.2-100% versus 80-100% and specificity of 99.5-100% versus 95.1-100%, but have rarely been evaluated in sub-Saharan Africa [3]. Oral fluid-based self-tests have achieved good sensitivity (93.6–100%) and specificity (99.1–100%) [15,16,19,39] by rural and urban Africans with a demonstration or provider supervision. In a recent blood-based study in South Africa, 96.4% of 224 participants correctly performed the self-test and interpreted the results under direct assistance [36^▪▪^]. Studies evaluating unassisted oral fluid-based self-tests have attained sensitivity of 66.7–90.0% and specificity of 95.2–100% [19,22,29]. Performance can depend on literacy level and previous exposure to HIV testing, but can be optimized through a demonstration-of-use [40–42]. Across studies, self-testing was often described as easy, with few reported errors [6,15,19,20,22,29,35^▪▪^,^36^^▪▪^,^38^,^39^,^43^]. Common missteps included incorrect specimen collection and use of the buffer solution and early reading or misinterpretation of results [15,19,29,36^▪▪^,^38^,^39^]. Visual stability of self-tests was inconsistent [44,45].

### Uptake of testing

Evidence on effective delivery models to increase testing coverage among underserved populations and testing frequency among high-risk populations is critical for informing HIVST implementation.

A foundational study in Malawi reported that community-based HIVST implementation led to high uptake, particularly among women and adolescents, in a high prevalence setting [16]. High demand for HIVST has been subsequently shown for pharmacy [34], facility [35^▪▪^,^43^] and partner-delivered [46^▪▪^,^47^^▪▪^] models. A cohort study in Kenya reported 98% uptake among 226 PrEP users who received self-tests in between clinic testing [35^▪▪^], showing high potential for HIVST to reduce the burden of PrEP on users and providers once more appropriate technologies for use among this population are available.

High uptake was also achieved under secondary distribution models. In a cohort of 280 pregnant and postpartum women and sex workers, 75–91% of participants across groups reported distributing kits to their primary partner and 80% of sex workers also distributed to commercial sex clients [47^▪▪^]. Among partners of sex workers who received a reactive result, 90% were clients, underpinning the ability of HIVST to reach high-risk individuals. A sister randomized controlled trial (*n* = 570) observed higher coverage of self-reported partner testing among pregnant and postpartum women when given HIVST kits for secondary delivery compared to partner invitation letters for clinic testing (90.8 versus 51.7%, *P* < 0.01) [46^▪▪^]. Couples testing and partner status disclosure were also more likely.

A number of ongoing randomized trials are examining the effectiveness of HIVST on recent and lifetime testing and positivity. The Self-Testing Africa (STAR) Initiative is conducting trials of community-based delivery of self-tests among the general population in Malawi (NCT02718274) and Zambia (NCT02793804). Other notable trials are evaluating direct and secondary distribution among young women and sex workers (NCT02827240; NCT02846402) and their partners (NCT03135067; NCT03162965).

### Linkage to prevention and care

Scalable strategies for confirmation of HIV status and continuation into the HIV prevention or care cascade are needed to maximize individual and public health benefits of HIVST. Studies have described suboptimal linkage to care following reactive results, though they were not designed to assess nor statistically powered on linkage [16,46^▪▪^,^47^^▪▪^,^48^]. In Malawi, linkage to care was 56.3% among community-based self-testers [16]. A study in Kenya reported linkage to confirmatory HTS was two of four among partners of pregnant and postpartum women and 26 of 41 among partners of sex workers [47^▪▪^]. In a Kenyan trial, two of eight sexual partners linked to care in the HIVST arm compared to three of four partners in the clinic testing arm, as reported by proxy [46^▪▪^].

To enhance linkage after self-testing, an early trial in Malawi found that the offer of home-based confirmatory testing and antiretroviral therapy (ART) initiation led to a three-fold increase in population-level ART demand compared to referral to facility-based care [48]. Interventions to facilitate timely linkage to care are also being investigated in Zimbabwe (PACTR201607001701788) and Malawi (ISRCTN18421340), with preliminary results from the latter study reporting significant benefits on linkage to VMMC and ART using financial and nonfinancial incentives [49].

### Cost and cost-effectiveness

Cost and cost-effectiveness estimates, which are highly context-specific and dependent on the delivery model and prevalence of undiagnosed HIV, are vital to inform national HIVST policy and implementation. Mathematical modeling from Zimbabwe suggests that HIVST has potential to be cost-effective, contingent on delivery to high-burden settings with low coverage of HIV testing; reductions in delivery costs through less resource-intensive implementation and cuts in HIVST unit costs; and improvements in linkage to prevention among HIV-negative individuals [50]. More epidemiological and economic data from HIVST programs are needed to parameterize cost-effectiveness estimates at a national level [51].

Ongoing trials mentioned in this review are aiming to address this evidence gap. Cost data are available from previous research in urban Malawi, with evidence that community-based HIVST has potential to be cost-effective in high-burden settings [52]. The mean cost per individual tested through community-based HIVST (US$8.78) was comparable to facility-based HTS (US$7.54) [53^▪▪^], but higher per HIV-positive individual identified (US$97.50 versus US$25.30-US$76.14). Self-testers incurred almost no costs, compared to US$2.93 for facility testers, and were diagnosed at earlier stages. A follow-on analysis reported no differences in economic and quality of life outcomes between self-testers and facility testers one year after ART initiation [54]. Maintaining a strong focus on implementation design to maximize cost-effectiveness will be important, especially as the prevalence of undiagnosed HIV declines.

### Social and behavioral impact

Broader social and behavioral effects of HIVST, specifically around social harms and sexual risk-taking, are important to evaluate and consider for scaled implementation. Reassuringly, the incidence of serious social harms related to HIVST has been uniformly low, with no cases of suicide and few reports of intimate partner violence to date [16,18,35^▪▪^,^46^^▪▪^,^47^^▪▪^]. Coercive testing has been reported by couples but highlights a complex dynamic, where pressure to self-test is balanced by the sense that primary partners are entitled to know each other's status [18]. Evidence on sexual risk-taking is limited, with one study in Kenya finding that a cohort of prepartum and postpartum women and female sex workers were significantly less likely to have sexual intercourse (18 versus 62%, *P* < 0.01) and more likely to use condoms (100 versus 44%, *P* < 0.01) if their partners received reactive compared to nonreactive self-test results [47^▪▪^]. Ensuring that self-testers are able to assess and avoid social harms and understand risks of serosorting, as well as developing systems for identification and management of social harms, are key considerations for implementers.

### Research gaps and priorities

Recent evidence in sub-Saharan Africa has established the feasibility, acceptability, accuracy and safety of HIVST among target populations, but has also revealed gaps that require immediate prioritization (Table 3). Most studies have taken place in southern and eastern Africa, with geographic diversification from west and central Africa needed. There is also limited evidence on blood-based self-tests, which use less costly specimen collection devices and are more sensitive compared to oral fluid-based tests, and are therefore vital to facilitate price reductions in HIVST products and for use among high-risk populations. Research on how implementers can improve HIVST performance by optimizing IFUs and developing clear support materials through systematic assessment and adaptation remains a priority across products.

**Table 3:** Progress and gaps toward scaling up HIV self-testing in sub-Saharan Africa

	Progress	Gaps
Technology	Four BSTs and three OFSTs available in SSA (second and third generation).LMIC public sector prices at US$2–3, with price reduction under charitable agreement. Private sector prices at US$8–16	Limited product innovation beyond IFU and package modification and single-use parts.Limited availability of more sensitive HIVST products in LMICs for high-risk populations. High pricing for LMIC markets
Policy	Release of WHO HIVST guidelines.15 SSA countries with supportive HIVST policies	Absence of supportive HIVST policies in remaining countries, especially in west and central Africa.Most countries do not have complete HIVST operational guidelines for scale-up
Regulation	One OFST product approved by WHO PQ.Two countries with international standard products available in limited private sector channels	No BST product approved by WHO PQ.Need for robust regulatory and postmarket surveillance system given potential private sector outlets
Evidence	High feasibility, acceptability and accuracy of HIVST across a wide range of delivery models and populations.Minimal cases of social harm.Emerging evidence on effectiveness of HIVST on increased testing coverage and demand for follow-on HIV services	More evidence needed on effectiveness and cost-effectiveness under multiple delivery models, including unrestricted distribution through public and private sectors and strategies to minimize linkage delays. Limited studies on BST and from west and central AfricaSecondary effects of HIVST, including on sexual risk-taking and healthcare efficiency, are unknown

BST, blood-based self-testing; HIVST, HIV self-testing; LMIC, low-income and middle income; OFST, oral fluid-based self-testing; PQ, prequalification; SSA, sub-Saharan Africa.

The majority of current evidence comes from small-scale observational studies, with findings emerging from a number of ongoing randomized trials and economic evaluations. Evidence on effective and affordable HIVST delivery models for increasing testing coverage among underserved populations and testing frequency among high-risk populations are necessary for country decision-making. Recent and ongoing trials have largely evaluated community-based and partner-delivered HIVST, revealing a gap among a wider range of distribution models. With scale-up in mind, unrestricted distribution through public and private sectors, as well as interventions to minimize linkage delays among hard-to-reach populations, should be evaluated. Studies should also ensure alignment of epidemiological and economic metrics with inputs required for mathematical modeling to inform national cost-effectiveness estimates, with the aim of effectively and efficiently reaching undiagnosed PLHIV. Finally, secondary effects of HIVST, including sexual risk-taking and potential for efficiency gains for providers through task-shifting, are relatively unknown and require further investigation.

## CONCLUSION

HIVST is moving towards scaled implementation, with the release of WHO guidelines, WHO prequalification of the first HIVST product, price reductions of HIVST products and a growing product pipeline. Multicountry evidence from southern and eastern Africa confirms high feasibility, acceptability and accuracy across many delivery models and populations, including adolescents, men and female sex workers, with reassuringly minimal harms. Evidence on the effectiveness of HIVST on increased testing coverage is strong, while evidence on demand generation for follow-on HIV prevention and treatment services and cost-effective delivery is emerging. Despite these developments, HIVST delivery remains limited outside of pilot implementation.

Important technology gaps include increasing availability of more sensitive HIVST products in LMICs. Regulatory and postmarket surveillance systems for HIVST also require further development. Randomized trials evaluating the effectiveness and cost-effectiveness under multiple distribution models, including unrestricted delivery and with a focus on linkage to HIV prevention and treatment, remain priorities. Diversification of studies from west and central Africa and around blood-based products should be addressed.

## Acknowledgements

We thank Cheryl Johnson from the World Health Organization for her comments.

### Financial support and sponsorship

The authors are supported by Unitaid (grant number: PO#8477–0–600). A.T.C. is supported by the Wellcome Trust (grant number: WT105828). E.L.C. is supported by the Wellcome Trust (grant number: WT091769).

### Conflicts of interest

There are no conflicts of interest.

## REFERENCES AND RECOMMENDED READING

Papers of particular interest, published within the annual period of review, have been highlighted as:

▪ of special interest
▪▪ of outstanding interest
